# Preoperative Non-Invasive Mapping for Targeted Concomitant Surgical Ablation of Non-Paroxysmal Atrial Fibrillation (PreMap Study)

**DOI:** 10.3390/jcm14020481

**Published:** 2025-01-14

**Authors:** David Santer, Brigitta Gahl, Ali Dogan, Florian Bruehlmeier, Ulisse Camponovo, Rory Maguire, Larissa Goldiger, Vanessa Boss, Nicole Weber, Lena Schmuelling, Stefan Gherca, Jens Bremerich, Nadine Cueni, Luca Koechlin, Michael Kühne, Jules Miazza, Oliver Reuthebuch, Alexa Hollinger, Martin Siegemund, Christian Sticherling, Friedrich Eckstein, Simon A. Amacher

**Affiliations:** 1 Department of Cardiac Surgery, University Hospital Basel, 4031 Basel, Switzerland; 2 Medical Faculty of the University of Basel, 4056 Basel, Switzerland; 3 Center for Biomedical Research and Translational Surgery, Medical University of Vienna, 1090 Vienna, Austria; 4 Department of Radiology, University Hospital Basel, 4031 Basel, Switzerland; 5 Intensive Care Unit, University Hospital Basel, 4031 Basel, Switzerland; 6 Cardiovascular Research Institute (CRIB), Department of Cardiology, University Hospital Basel, 4031 Basel, Switzerland; 7 Department of Anesthesiology and Intensive Care Medicine, Medical Center–University of Freiburg, Faculty of Medicine, University of Freiburg, 79106 Freiburg im Breisgau, Germany

**Keywords:** cardiac surgery, surgical ablation, non-invasive mapping, atrial fibrillation, Cox-Maze

## Abstract

**Background/Objectives**: The present study introduces our targeted approach for concomitant surgical ablation (CSA) using non-invasive phase mapping (NIPM) and describes its effectiveness regarding freedom from atrial fibrillation (AF). **Methods**: This retrospective study included cardiac surgical patients undergoing preoperative NIPM for CSA guidance. The primary outcome was freedom from AF six months after surgery. Key secondary outcomes were freedom from AF at hospital discharge and three months, frequency of biatrial ablation, feasibility and safety, the rate of CSA, complications, and levels of biomarkers. The control group consisted of patients undergoing CSA without NIPM. **Results**: Forty-four patients (Control: *n* = 31/NIPM: *n* = 13) were included. The NIPM group was younger (64 vs. 71 years [*p* = 0.044]), had a lower EuroSCORE II (2.6 vs. 3.4 [*p* = 0.041]), and a smaller left atrial size (46 mm vs. 54 mm [*p* = 0.025]). Surgery duration was longer in the NIPM group (285 vs. 230 min [*p* = 0.037]) with similar aortic cross-clamp times. Preoperative NIPM resulted in an effective frequency of CSA of 93%. CSA was more extensive in the NIPM group, with biatrial ablation performed in 54% vs. 26% of patients (*p* = 0.09). **Conclusions**: Routine preoperative NIPM in patients with non-paroxysmal atrial fibrillation might aid in increasing the number of patients receiving concomitant surgical ablation and developing a personalized CSA approach for every patient.

## 1. Introduction

Concomitant surgical ablation (CSA) in patients with atrial fibrillation (AF) is defined as surgical ablation of atrial fibrillation when performing cardiac surgery for other reasons (e.g., valvular surgery or coronary artery bypass grafting) [1,2,3]. CSA is known for its excellent long-term results, with freedom from AF of 91% after one, 84% after five, and 77% after 10 years [4]. In the 2024 ESC Guidelines, CSA carries a class I indication in patients undergoing mitral valve surgery and class IIa indication for all other types of cardiac surgery [5]. Nevertheless, CSA is only performed in 7.7–22.2% of AF patients during cardiac surgery [6,7], with high-volume centers reporting higher proportions [8]. Potential reasons for not performing CSA are prolonged aortic cross-clamp times, technical complexity, and doubts regarding efficacy [9,10]. Worldwide, there is great variation in surgical ablation strategies ranging from single lesions to extensive biatrial ablation, i.e., Cox-Maze IV procedure [11]. Thus, there is a need for a more personalized and patient-centered surgical ablation approach.

Non-invasive phase mapping (NIPM) is a promising tool that allows the characterization of AF using a 252-electrode vest applied to a patient’s torso [12]. Together with a low-dose computed tomography scan, it provides a detailed map of AF-maintaining structures in the individual patient without the need for an invasive electrophysiological examination [13]. With NIPM, a biatrial roadmap of rotors and foci is created [14], which might allow a more targeted surgical ablation strategy. NIPM has previously been suggested as a planning tool to analyze AF-maintaining rotors and foci preoperatively, allowing a more personalized surgical ablation strategy [14]. Next to its application for AF, NIPM has been found to be effective in the risk assessment of ventricular tachycardias [15], in the mapping of ventricular tachycardias, for the planning of stereotactic arrhythmia radioablation [12], and as an outcome prediction tool in cardiac resynchronization therapy (CRT) [16].

In this study, we used NIPM in the clinical setting as a novel strategy to guide CSA in patients undergoing cardiac surgery. For the first time, we tested the feasibility of this approach in a cohort of patients with non-paroxysmal AF undergoing cardiac surgery.

## 2. Materials and Methods

### 2.1. Study Design

**Figure 1:**
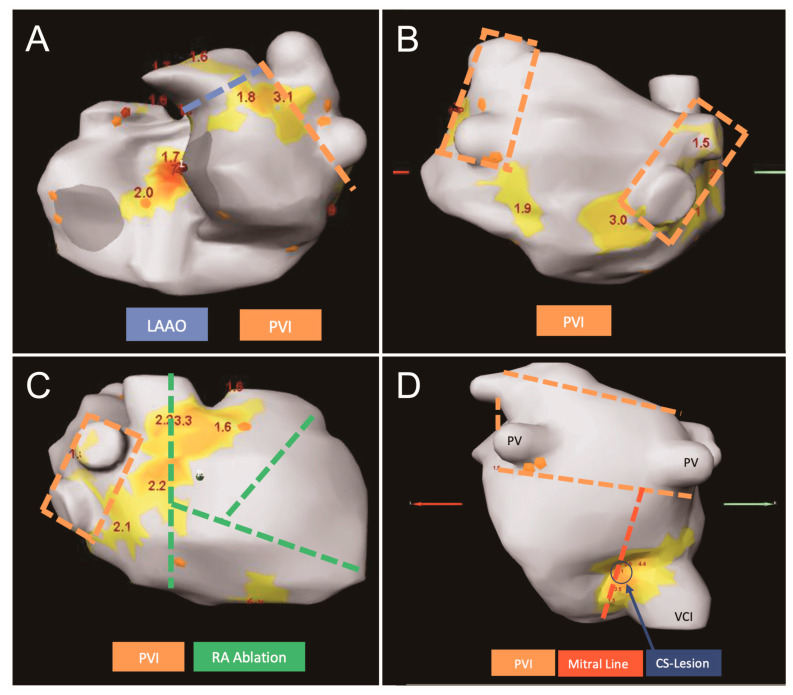
Sample of suggested lesion sets of 2 patients (patient 1: **A**–**C**; patient 2: **D**) provided to the surgeon. Single lesions from the Cox-Maze IV lesion set were chosen to target identified rotors. (**A**): left lateral view, (**B**): posterior–anterior view, (**C**): right lateral view, (**D**): posterior–anterior view. CS: coronary sinus (blue arrow), LAAO: left atrial appendage occlusion (blue line), Mitral Line (red line), PVI: pulmonary vein isolation (orange lines), RA: right atrial (green lines).

This retrospective single-center cohort study included patients (≥18 years) undergoing elective cardiac surgery with preoperative non-paroxysmal AF between February 2015 and May 2023. In 2020, NIPM (Cardiolnsight^®^, Medtronic Switzerland, Tolochenaz, Switzerland) was introduced as a clinical pilot project at our institution. All patients with non-paroxysmal AF who agreed to undergo CSA were invited for preoperative NIPM (NIPM group, Figure 1). The NIPM group was then retrospectively compared to a control group, which consisted of all consecutive patients with non-paroxysmal AF undergoing CSA without preoperative NIPM. Patients who declined consent were excluded. Patient characteristics, risk factors, surgical details, and outcome data are routinely collected in the department’s prospectively maintained quality management software (V1.7, Dendrite Clinical Systems, Reading, UK), and regularly checked for completeness and consistency. Follow-up data (periodic 12-channel ECG or Holter ECGs) were collected three and six months after surgery by contacting the referring physicians.

### 2.2. Outcomes

The primary outcome was defined as freedom from AF six months after surgery. Freedom from AF was defined according to the rhythm extracted from follow-up data (periodic 12-channel ECG or Holter ECG). Key secondary outcomes were freedom from atrial fibrillation at hospital discharge and three months, frequency of biatrial ablation, and the rate of CSA in both groups. As additional secondary outcomes, postoperative complications (reoperation for bleeding, sepsis/infections, postoperative myocardial infarction, postoperative stroke, renal replacement therapy, operative mortality, permanent pacemaker implantation) and biomarkers (creatine kinase, high-sensitivity troponin T, creatinine) were assessed.

### 2.3. Mapping Procedure

Between 2020 and 2023, all candidates for cardiac surgery with non-paroxysmal AF were invited to undergo a preoperative NIPM in our outpatient clinic. A Cardiolnsight^®^ (CIT) cardiac mapping vest (Medtronic Switzerland, Tolochenaz, Switzerland) was applied to patients as part of the mapping procedure. CIT is a non-invasive single-beat cardiac phase mapping system that provides three-dimensional electroanatomic heart maps indicating AF’s potential drivers (focal and rotational activity) [17,18]. In patients with a heart rate > 50 bpm, it is necessary to briefly lower the heart rate, thereby avoiding ventricular potentials to superimpose over atrial potentials. To achieve this, a single 6–18 mg rapid intravenous bolus of adenosine (Krenosin^®^, Sanofi-Aventis [Suisse] SA, Vernier, Switzerland) was administered to patients under hemodynamic monitoring. Consecutively, a low-dose chest computed tomography (CT) scan (neck to upper abdomen) with the patient still wearing the CIT vest was performed. The CT scan followed a standardized protocol predefined by the CIT vest manufacturer. If extended preoperative CT imaging was indicated for any other reason, an extended CT scan (ECG-gated, contrast agent, etc.) was performed instead of a low-dose CT, which did not limit the reliability of the NIPM analysis.

**Figure 2:**
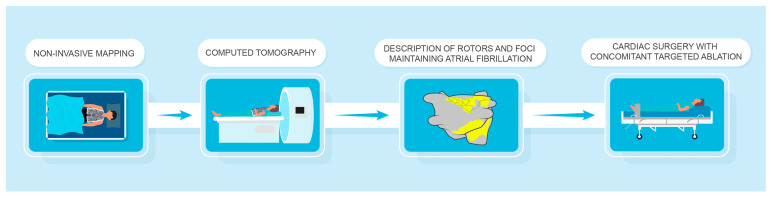
Patients with non-paroxysmal atrial fibrillation underwent non-invasive mapping prior to cardiac surgery. Follow-up was performed at hospital discharge, 3 months, and 6 months after surgery.

After NIPM, a protocol with a suggested lesion set according to NIPM was provided to the surgeon. Lesions from the Cox-Maze IV lesion set were chosen for any rotational activity with at least two rotations during recording. Rotational activity with less than two rotations was not considered significant. Patients without NIPM underwent CSA according to the surgeon’s preference. Figure 2 provides an overview of the study and mapping procedure.

**Figure 3:**
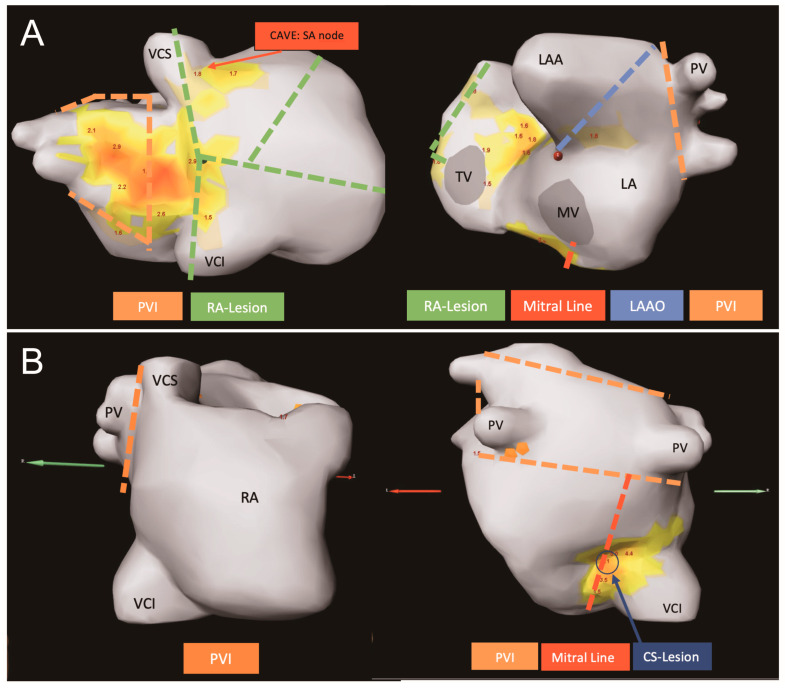
Biatrial (horizontal Panel **A**, left: right lateral view, right: left lateral view) and left atrial approach (horizontal Panel **B**, left: right lateral view, right: posterior–anterior view). PVI: pulmonary vein isolation (orange lines), RA: right atrial ablation (green lines), LAAO: left atrial appendage occlusion (blue line), CS: coronary sinus (blue arrow), Mitral Line (red line), LA: left atrium, LAA: left atrial appendage, MV: mitral valve, PV: pulmonary vein, RA: right atrium, SA: sinoatrial, TV: tricuspid valve, VCI: inferior vena cava, VCS: superior vena cava.

A sample of a suggested lesion set is shown in Figure 1. Hereby, we describe two exemplary patients:

Patient A was referred for severe aortic stenosis and non-paroxysmal AF. NIPM showed biatrial rotational activity; therefore, aortic valve replacement and Cox-Maze IV cryoablation with epicardial occlusion of the left atrial appendage (Atriclip Flex V, AtriCure Inc., Mason, OH, USA) were performed (Figure 3, Panel A).

Patient B was referred for severe mitral valve regurgitation and non-paroxysmal AF. NIPM revealed rotational activity in the coronary sinus region and focal activity below the left inferior pulmonary vein. Therefore, the patient underwent minimal invasive mitral valve repair and endocardial left atrial cryoablation with suturing of the left atrial appendage performed. No right atrial ablation was performed as no potential AF drivers were identified in this region (Figure 3, Panel B). 

### 2.4. Postoperative Management

Postoperative management after CSA was performed according to institutional guidelines: all patients received amiodarone 600 mg/d for 20 days, followed by amiodarone 200 g/d for three months. Oral anticoagulation was continued in all patients independent from postoperative rhythm.

### 2.5. Ethics

This study complies with the Declaration of Helsinki, and the study protocol has been approved by the local Ethics Committee of Northwestern and Central Switzerland on 5 January 2024 (Project-ID 2022-00101).

### 2.6. Statistical Methods

To investigate whether preoperative mapping was associated with longer postoperative sinus rhythm (SR) periods, we used multilevel logistic models, including SR/AF during follow-up as the dependent variable, time and patient risk factors as fixed effects, and patient identifiers as random effects. SR or AF during follow-up was assumed to last from diagnosis until diagnosis of the opposite. Due to the small sample size, we refrained from adjustments for known influence factors. LA diameter was missing in 16 patients, which was accounted for with a mean imputation method. We visualized observed periods of SR and AF, respectively, and interventions to support SR as a lineplot. We refrained from applying propensity modelling or other methods to balance the risk of non-ablation among treatment groups given the small group sizes which do not allow for the necessary assumption checks.

We summarized continuous variables as median with interquartile range and categorical variables as numbers with percentages. Comparisons of groups were substantiated using Wilcoxon–Mann–Whitney’s test or Fisher’s exact test, respectively. The ablation grade was defined by the extent of the performed ablation; in other words, the ablation grade correlated with the number of ablation lines.

All analyses were carried out by a trained statistician (B.G.) using Stata 16 (Stata Corp LLC, Lakeway Drive, College Station, TX, USA).

## 3. Results

### 3.1. Patient Characteristics

**Figure 4:**
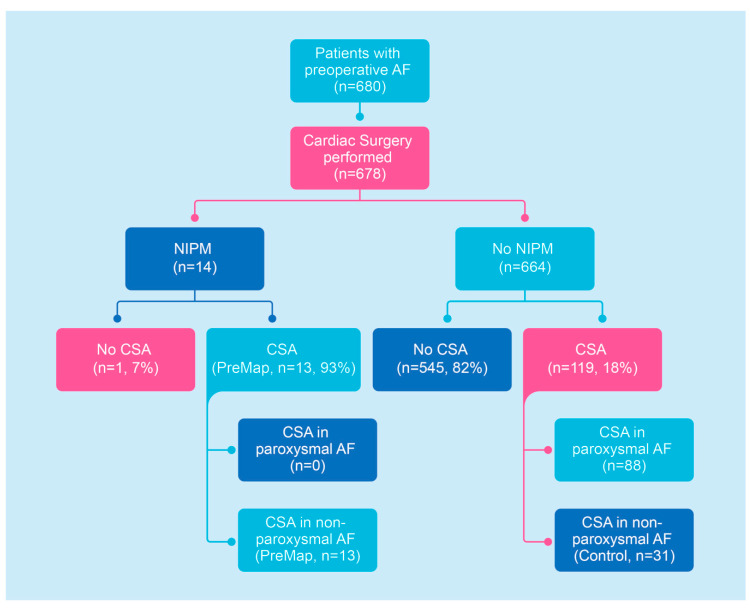
Study flow chart. Abbreviations: AF—atrial fibrillation, CSA—concomitant surgical ablation, NIPM—non-invasive preoperative mapping.

From 2015 to 2023, 680 patients with preoperative AF (paroxysmal and non-paroxysmal) were referred for cardiac surgery. In two of these patients, NIPM was performed, but surgery was omitted since one patient died due to a non-cardiac disease, and one patient declined to undergo cardiac surgery before admission. Of the final 678 patients, 14 patients with non-paroxysmal AF underwent NIPM and consecutive cardiac surgery. In one of these patients, no CSA was performed (surgeon’s decision), which resulted in exclusion from further analysis. Finally, 13 patients (93%) underwent CSA after successful NIPM. Of 664 AF patients (non-paroxysmal and paroxysmal) who did not receive preoperative NIPM, 119 patients (88 paroxysmal AF, 31 non-paroxysmal AF) underwent CSA. Finally, 31 patients (Control) with non-paroxysmal AF undergoing CSA without NIPM and 13 patients with NIPM (NIPM group) were included in the final analysis (Figure 4).

The NIPM group was younger (*p* = 0.04) and had a lower EuroSCORE II (*p* = 0.04) and smaller LA size (*p* = 0.019); all other preoperative characteristics were similar. NIPM was successfully performed in all patients. No complications during NIPM were observed (Table 1).

### 3.2. Surgical Details

All patients underwent elective surgery. The two groups did not differ regarding the type of procedures, with the most common surgery being isolated mitral valve surgery (NIPM vs. Control: 62 vs. 68%). Duration of surgery was longer in the NIPM group (285 [222 to 298] vs. 230 [190 to 273] min; *p* = 0.037), but aortic cross-clamp times were similar. There was no statistical difference in the performed lesion sets between the two groups, as can be seen in Table 2.

### 3.3. Postoperative Outcomes

**Figure 5:**
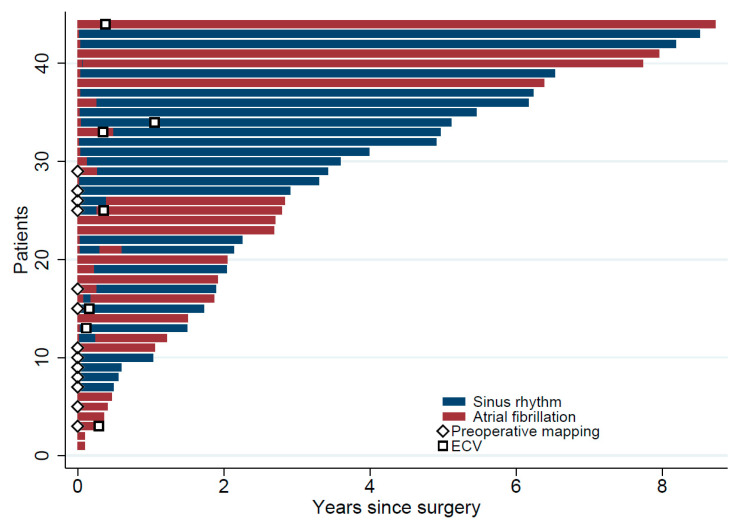
Observed sinus rhythm, atrial fibrillation periods, and electrocardioversion (ECV) as a lineplot. Each horizontal line represents each patient’s observed rhythm (blue: sinus rhythm, red: atrial fibrillation) during follow-up. Patients with preoperative mapping are marked with a tilted white square. The time points of electrocardioversion are indicated with (non-tilted) white squares.

No severe postoperative complications were observed in either group. Postoperative cardiac biomarkers and freedom from AF were similar at discharge (NIPM vs. Control: 62 vs. 58%; *p* = 1.0), after three months (77 vs. 58%; *p* = 0.31), and after six months (NIPM vs. Control: 77 vs. 74%; *p* = 1.0, Figure 5 and Table 3).

## 4. Discussion

To the best of our knowledge, this is the first proof-of-concept study describing the feasibility and safety of NIPM for targeted CSA. In total, thirteen elective patients with non-paroxysmal AF underwent preoperative NIPM and targeted CSA according to a provided mapping protocol. Thirty-one patients with non-paroxysmal AF (control group) underwent CSA according to each surgeon’s preference. The use of NIPM showed no difference observed in the rate of AF at discharge, three months, and six months after surgery, and safety endpoints. However, surgical ablation was more extensive in the NIPM group, with biatrial ablation performed in 54% vs. 26% (*p* = 0.09) of patients.

Arrhythmia-free survival ranges from 47 to 75% after interventional pulmonary vein isolation in persistent atrial fibrillation [19]. In mitral and non-mitral cardiac surgery in patients with persistent AF, the Cox-Maze IV (CM) procedure is superior to pulmonary vein isolation (PVI) alone in freedom from AF at 12 months (75 vs. 47%) [20,21]. In a small study of NIPM in 10 patients with persistent long-standing AF, biatrial potential drivers of AF were observed in all patients. Also, Osorio-Jaramillo and colleagues observed biatrial rotors and foci in patients with long-standing persistent AF before cardiac surgery [22]. Therefore, the authors of both studies suggested a complete Cox-Maze IV procedure when performing CSA [14,22].

Although the Cox-Maze procedure is the gold standard in surgical ablation [23], its benefits are still unclear compared to sole LA ablation. While the 2020 ESC Guidelines have recommended biatrial over LA concomitant ablation in patients with non-paroxysmal AF [23], the recent 2024 ESC Guidelines do not give a distinct recommendation [5]. However, in mitral valve surgery, biatrial ablation bears a risk of 25% for postoperative pacemaker implantation [24,25]. A meta-analysis analyzed the outcomes of LA vs. biatrial ablation and showed comparable short and long-term mortality. Freedom from AF was better in biatrial patients after one year but not beyond. Complications rates were similar, except for a higher pacemaker implantation rate after biatrial ablation [26]. In our study, we could not confirm a biatrial mechanism of AF in all patients, as suggested by Ehrlich et al. [14]; therefore, full CM was only performed in 54% of cases. While the box lesion as part of LA ablation plays a key role in CSA success, the benefits of a biatrial lesion set versus left-atrial ablation only are still under debate [26]. Freedom from AF is comparable with 73 vs. 78% five years after biatrial vs. left-sided ablation [27]. However, biatrial ablation, i.e., more extensive ablation, is associated with a risk of permanent pacemaker implantation [4,25,26,28].

Interestingly, the rate of biatrial ablation was still higher than in the control group, which might be an effect of the targeted approach. Of interest, the rate of postoperative pacemaker implantations was similar between the NIPM and the control group. Although CM was not performed in all NIPM patients, freedom from AF was satisfactory, with 77% after six months, similar to other studies [14,29]. In addition, our study showed no difference in recurrence of AF whether NIPM was performed or not.

Left atrial appendage closure was associated with reduced AF recurrence at discharge and three months but not at six months, which might highlight the LAA’s electrical importance in addition to its thrombogenic role. The ablation grade showed a borderline correlation with AF at discharge and overall. This might be due to patients with a higher density of potential drivers demanding a more extensive lesion set and, simultaneously, having a higher risk for the recurrence of AF.

Although the existing literature indisputably favors CSA, its frequency is generally low. Niv et al. showed that CSA did not increase procedure-related complications but improved survival [8]. However, the same study also observed a prolonged ICU and in-hospital stay duration in patients after CSA [8]. A reasonable argument to avoid CSA seems to be the size of the left atrium, which our data also confirmed as a predictor for the recurrence of AF. Interestingly, in 759 patients with giant left atria, the CM procedure was beneficial in terms of freedom from AF (69 vs. 10%) after 5 years, lower rate of thromboembolic events (HR 0.23), and even death (HR 0.68) [30]. Younger patients with giant LA might even benefit more from CSA. While data show that LA size should not prohibit CSA, 86% of surgeons agreed that the size of the LA influenced their decision to perform CSA [9]. Thus, in our study, this new technology was offered to all patients with non-paroxysmal AF independent of obvious risk factors.

In a nationwide study from Taiwan, high-volume centers had better outcomes after CSA than low-volume centers [31]. Noteworthy, only 52% of surgeons felt influenced if the referring cardiologist recommended CSA. In this pilot study, we showed that NIPM increased the rate of CSA in patients with non-paroxysmal AF to 93%, while patients without previous NIPM received CSA in 18% of cases. The high acceptance rate might also be explained by the fact that NIPM was performed by surgeons for surgeons (“within the same team”). Our results align with the recent literature’s recommendation on strategies to increase awareness for AF therapy, e.g., by standardized protocols [32]. Although chronic underutilization of CSA has been shown in register studies [33], to the best of our knowledge, no distinctive strategies have been proposed yet to increase awareness for CSA in cardiac surgery [32]. If needed for further diagnosis, the NIPM-CT scan was performed with contrast agent and/or ECG-gated, so that radiation exposure was limited. Of note, in one patient, the preoperative NIPM-CT scan revealed unknown COVID-19 infection and, therefore, urged us to postpone surgery [13]. In our opinion, the efforts needed to run an NIPM program were limited, while NIPM increased the awareness of patients and surgeons [32]. Our preoperative NIPM program was performed using a standardized protocol. This resulted in a higher CSA frequency than reported by high-volume centers [8].

Increased case numbers necessarily lead to better results with a higher rate of complete lesion sets [31]. It seems evident that knowledge of the location of potential drivers of AF might allow a more targeted surgical ablation. The results of this study show that biatrial ablation might not be necessary in all patients with non-paroxysmal AF and, therefore, supports the concept of limiting CSA to the structures that are easily accessible during primary surgery [23]. NIPM might further support the discussion on the necessity of biatrial ablation since existing data are controversial. Next to the discussion about the extent of CSA, more light should be shed on the underusage of CSA in general. The effect of NIPM on the frequency of CSA might hint at how to address this issue, be it with NIPM, dedicated arrhythmia teams, or any other efficient strategy to increase awareness for this beneficial therapy.

### Limitations

This study’s limitations are its single-center design, retrospective nature, and small sample size. The groups showed differences in important patient characteristics, such as LA size, EuroSCORE II, and age, which might have influenced the results. Due to the small sample size, the underlying disease of AF and the energy source used for ablation were not included in the analysis. Also, the small sample size might have resulted in insufficient power to detect differences between groups. However, the primary aim was to propose a novel approach to CSA. The benefit of this approach will have to be proven in larger multi-centric studies. Additionally, the availability of NIPM is still limited in most cardiac surgical departments, and reimbursement of NIPM might vary in each country. Reasons for the high costs of NIPM are the single-use vests and the continuous need for technical support, especially in the beginning of an NIPM program. Nevertheless, most systems are installed in electrophysiology labs and can be mounted on a mobile rack, so NIPM sharing is possible between cardiology and cardiac surgery.

## 5. Conclusions

Within this study, we propose NIPM as a new preoperative diagnostic tool for CSA in patients with non-paroxysmal AF. Using NIPM might allow a surgeon to develop a personalized ablation approach for each individual patient, potentially reducing the extensiveness of ablation, which might result in shorter aortic cross-clamp times, fewer postoperative complications, and improved overall outcomes. Due to the small sample size, the present study could not detect a difference between conventional CSA and NIPM-based CSA. In addition to this device’s diagnostic potential, our results underline the importance of awareness in achieving high rates of CSA. Future prospective studies should focus on tools to increase the frequency of CSA and further analyze whether a more precise and selective CSA approach based on NIPM can improve outcomes.

## Figures and Tables

**Table 1:** Patient characteristics. Continuous variables are presented as median and interquartile ranges. Abbreviations: EuroSCORE II—European system for cardiac operative risk evaluation II, LA—left atrium, MI—myocardial infarction, NIPM—non-invasive preoperative mapping, S—seconds.

	Total (*N* = 44)	Control (*N* = 31)	NIPM (*N* = 13)	*p*
Age, y	69 [64 to 74]	71 [66 to 75]	64 [62 to 69]	0.044
Female	8 (18%)	5 (16%)	3 (23%)	0.68
Diabetes mellitus	5 (11%)	4 (13%)	1 (8%)	0.33
Peripheral artery disease	5 (11%)	4 (13%)	1 (7.7%)	1.00
Preoperative Stroke	7 (16%)	3 (10%)	4 (31%)	0.17
Renal disease	3 (6.8%)	2 (6.5%)	1 (7.7%)	1.00
Dialysis	0 (0%)	0 (0%)	0 (0%)	
Prior MI	2 (4.5%)	1 (3.2%)	1 (7.7%)	0.51
Hypertension	0 (0%)	0 (0%)	0 (0%)	
Hypercholesteremia	0 (0%)	0 (0%)	0 (0%)	
Current smoker	0 (0%)	0 (0%)	0 (0%)	
Ejection fraction, %	56 [50 to 60]	55 [48 to 60]	58 [55 to 60]	0.28
EuroSCORE II	3.3 [1.9 to 4.8]	3.4 [2.2 to 6.2]	2.6 [1.5 to 2.9]	0.041
LA size. mm	50 [45 to 56]	54 [48 to 57]	46 [41 to 49]	0.025
CHA2DS2-VASc	2.0 [1.0 to 2.0]	2.0 [1.0 to 2.0]	1.0 [0.00 to 2.0]	0.41
NIPM				
Successful examination		-	13 (100%)	
Procedure-related complications		-	0 (0%)	-
Length of mapping, ms		-	13,700 [10,228 to 15,572]	-
Patients with focal activity		-	13 (100%)	-
Number of focals			5 [4 to 6]	-
Cumulative number of focals		-	62	-
Patients with rotor activity			13 (100%)	-
Cumulative number of rotors		-	494	-
Number of rotors			37 [25 to 45]	-

**Table 2:** Surgical data. Continuous variables are presented as median and interquartile ranges. Abbreviations: CABG—coronary artery bypass grafting, LAA—left atrial appendage, NIPM—non-invasive preoperative mapping.

	Total (*N* = 44)	Control (*N* = 31)	NIPM (*N* = 13)	*p*
CABG only	5 (11%)	4 (13%)	1 (7.7%)	1.00
CABG and Valve(s)	10 (23%)	9 (29%)	1 (7.7%)	0.24
Valve(s) only	28 (64%)	17 (55%)	11 (85%)	0.09
Aortic valve	13 (30%)	9 (29%)	4 (31%)	1.00
Mitral valve	29 (66%)	21 (68%)	8 (62%)	0.74
Tricuspid valve	9 (20%)	8 (26%)	1 (7.7%)	0.24
Combinations				0.65
Single valve	26 (59%)	15 (48%)	11 (85%)	0.043
Double/triple valve	12 (27%)	11 (35%)	1 (7.7%)	0.07
Aortic replacement *	4 (9.1%)	2 (6.5%)	2 (15%)	0.57
Removal of LAA Tumor	1 (2.3%)	1 (3.2%)	0 (0.00%)	1.00
Tricuspid insufficiency				0.17
No	32 (73%)	21 (68%)	11 (85%)	
low or moderate	5 (11%)	3 (10%)	2 (15%)	
Severe	7 (16%)	7 (23%)	0 (0.00%)	
Duration of surgery, min	240 [192 to 286]	230 [185 to 273]	285 [222 to 298]	0.037
Aortic cross-clamp time, min	103 [89 to 124]	103 [86 to 119]	103 [96 to 133]	0.23
Lesion Sets				
Left atrial ablation	29 (66%)	24 (74%)	6 (46%)	0.09
Biatrial ablation	15 (34%)	8 (26%)	7 (54%)	0.09
LAA closure	32 (73%)	21 (68%)	11 (85%)	0.46

* Concomitant replacement of ascending aorta and hemi-arch with aortic valve.

**Table 3:** Continuous variables are presented as median and interquartile ranges. Abbreviations: AF—atrial fibrillation, CK—creatine kinase, CK-MB—creatine kinase–muscle brain, MI—myocardial infarction, NIPM—non-invasive preoperative mapping.

	Total (*N* = 44)	Control (*N* = 31)	NIPM (*N* = 13)	*p*
Complications				
Reoperation for bleeding	0 (0%)	0 (0%)	0 (0%)	
Sepsis/Infection	0 (0%)	0 (0%)	0 (0%)	
Postoperative MI	0 (0%)	0 (0%)	0 (0%)	
Postoperative Stroke	0 (0%)	0 (0%)	0 (0%)	
Renal replacement therapy	3 (6.8%)	3 (10%)	0 (0%)	0.54
Operative mortality	0 (0%)			
Permanent pacemaker	14 (32%)	9 (29%)	5 (38%)	0.72
Biomarker				
Max. CK value	1201 [854 to 1573]	1078 [693 to 1512]	1438 [1198 to 1671]	0.07
Max. CK-MB value	75 [44 to 122]	73 [28 to 104]	97 [69 to 132]	0.09
Max. hsTroponin T, ng/L	1858 [1214 to 2810]	1792 [1180 to 2624]	2000 [1560 to 3266]	0.31
Max. Creatinine value (umol/L)	108 [92 to 150]	108 [93 to 161]	99 [81 to 141]	0.21
Hospital length of stay	10 [7.5 to 13]	9.0 [8.0 to 14]	12 [7.0 to 13]	0.96
Freedom from AF				
Discharge	26 (59%)	18 (58%)	8 (62%)	1.00
3 months	28 (64%)	18 (58%)	10 (77%)	0.31
6 months	33 (75%)	23 (74%)	10 (77%)	1.00

## Data Availability

The raw data supporting the conclusions of this article will be made available by the authors on request.
